# Augmenting propulsion demands during split-belt walking increases locomotor adaptation of asymmetric step lengths

**DOI:** 10.1186/s12984-020-00698-y

**Published:** 2020-06-03

**Authors:** Carly J. Sombric, Gelsy Torres-Oviedo

**Affiliations:** Department of Bioengineering, University of Pittsburgh, 4420 Bayard Street, Suite 110, Pitt, Pittsburgh, PA USA

**Keywords:** Stroke, Motor learning, Hemiparesis

## Abstract

**Background:**

Promising studies have shown that the gait symmetry of individuals with hemiparesis due to brain lesions, such as stroke, can improve through motor adaptation protocols forcing patients to use their affected limb more. However, little is known about how to facilitate this process. Here we asked if increasing propulsion demands during split-belt walking (i.e., legs moving at different speeds) leads to more motor adaptation and more symmetric gait in survivors of a stroke, as we previously observed in subjects without neurological disorders.

**Methods:**

We investigated the effect of propulsion forces on locomotor adaptation during and after split-belt walking in the asymmetric motor system post-stroke. To test this, 12 subjects in the chronic phase post-stroke experienced a split-belt protocol in a flat and incline session so as to contrast the effects of two different propulsion demands. Step length asymmetry and propulsion forces were used to compare the motor behavior between the two sessions because these are clinically relevant measures that are altered by split-belt walking.

**Results:**

The incline session resulted in more symmetric step lengths during late split-belt walking and larger after-effects following split-belt walking. In both testing sessions, subjects who have had a stroke adapted to regain speed and slope-specific leg orientations similarly to young, intact adults. Importantly, leg orientations, which were set by kinetic demands, during baseline walking were predictive of those achieved during split-belt walking, which in turn predicted each individual’s post-adaptation behavior. These results are relevant because they provide evidence that survivors of a stroke can generate the leg-specific forces to walk more symmetrically, but also because we provide insight into factors underlying the therapeutic effect of split-belt walking.

**Conclusions:**

Individuals post-stroke at a chronic stage can adapt more during split-belt walking and have greater after-effects when propulsion demands are augmented by inclining the treadmill surface. Our results are promising since they suggest that increasing propulsion demands during paradigms that force patients to use their paretic side more could correct gait asymmetries post-stroke more effectively.

## Background

Brain lesions, such as stroke, may result in asymmetric gait, limiting patients’ mobility and decreasing their quality of life [31]. Moreover, gait asymmetry can lead to comorbidities further affecting post-stroke gait such as musculoskeletal injuries [32] and joint pain [59]. Promising studies have shown that motor adaptation protocols forcing individuals to use their affected limb more, as in “constrained use therapy” [36], could lead to motor improvements. For example, split-belt walking (i.e., legs moving at different speeds), has been shown to reduce gait asymmetries post-stroke [7, 39–41, 47, 62, 63]. While this is encouraging, little is understood about the mechanisms underlying this process and how to facilitate it. This is relevant since not all individuals improve their gait following repeated exposure to split-belt walking [7, 61]. Therefore, there is a scientific and clinical interest to identify factors that underlie the therapeutic effect of split-belt walking in order to augment rehabilitative effects.

Our previous work indicates that locomotor adaptation in young, unimpaired subjects increases by augmenting propulsion demands during split-belt walking. More specifically, greater propulsion demands during incline split-belt walking resulted in greater changes in step lengths relative to flat split-belt walking [70]. Individuals post-stroke have well-known deficits in paretic propulsion [4, 9] leading to asymmetric step lengths [65]. This deficient force generation raise the question of whether survivors of a stroke could increase their paretic propulsion to walk in the incline split-belt condition, and in turn exhibit greater adaptation of step length asymmetry during and after split-belt walking, as we observed in young, intact individuals [70]. We consider that this would be a possibility since there is evidence that survivors of a stroke can augment their propulsion forces when required by the walking condition; for example, when walking at fast speeds [3, 24–26, 34]. Thus, we tested whether the adaptation of step length asymmetry in survivors of a stroke could be augmented by increasing propulsion demands with incline split-belt walking.

We hypothesized that increasing propulsion demands during the split-belt condition by inclining the walking surface, which naturally augments propulsion forces [42, 43], would lead to greater adaptation of step length asymmetry during split-belt walking and larger after-effects in individuals post-stroke. This was formulated on the basis of our results in young, unimpaired individuals [70]. To test our hypothesis, we performed a research study with subjects in the chronic phase post-stroke, who experienced two split-belt adaptation protocols with distinct propulsion demands: a flat configuration and an incline configuration. We expected more step symmetry adaptation and greater after-effects following incline split-belt walking relative to flat split-belt walking. We also anticipated that steady-state split-belt walking and the following after-effects from each individual could be predicted from the subject-specific baseline gait; more specifically, from their baseline feet positions relative to the body at foot landing (i.e., leg orientations) needed to walk at the speed and inclination set on each leg in the split condition [70]. Therefore, we expected that baseline walking would be predictive of the step lengths achieved during split-belt walking and after-effects in an individual basis. These anticipated findings would suggest that therapies increasing bilateral propulsion demands during walking would be a good strategy for improving post-stroke gait.

## Methods

We investigated the effect of augmenting propulsion demands during split-belt walking on gait adaptation under distinct slopes (i.e., flat and incline), which modulates propulsion forces [42, 43]. To this end, we evaluated the adaptation and after-effects of 12 patients who have had a stroke (4 females, 60.3 ± 10.0 years of age) in the chronic phase of recovery (> 6 months post-stroke) during separate flat and incline testing sessions. Those who have had a stroke were eligible if they (1) had only unilateral and supratentorial lesions (i.e., without brainstem or cerebellar lesion) as confirmed by MRI, (2) were able to walk without assistance from others or a device for 5 min at a self-selected pace, (3) were free of orthopedic injury or pain that would interfere with testing, (4) had no other neurological condition other than stroke, (5) had no severe cognitive impairments defined by a Mini-Mental State Exam score below 24 [55], and (6) did not take medications that altered cognitive function. Overall, participants that met the inclusion criteria were mildly to moderately impaired post-stroke [13], as indicated by their Lower Extremity Fugl-Meyer score and walking speed (Table 1). Participants gave written and informed consent prior to participation. The University of Pittsburgh Institutional Review Board approved the experimental protocol experienced by all participants.

**Table 1:** Clinical characteristics of stroke survivors

ID	Age	Gender	Affected Side	Lesion Location	Fugl-Meyer Score	Slow, Mid, Fast Speed (m/s)	Total # Adapt Strides (flat/ incline)	Total # Post Strides (flat/ incline)	Incline Session Slope (°)
P1	43	Female	R	Left MCA and basal ganglia	33	0.75 1.13 1.50	907/609	605/303	8.5°
P2	55	Female	R	Left MCA and ACA, temporal lobe, basal ganglia	26	0.54 0.81 1.08	867/301	642/300	5°
P3	64	Female	R	Left MCA, frontal, parietal lobe and basal ganglia	29	0.40 0.60 0.80	617/368	307/10	5°
P4	58	Female	R	Left medial, frontal and parietal area’s	21	0.30 0.45 0.60	901/406	625/10	5°
P5	66	Male	R	Left MCA, frontal, temporal and parietal lobes	30	0.51 0.77 1.02	606/452	599/302	5°
P6	60	Female	R	Left frontal	26	0.60 0.90 1.20	907/597	600/300	5°
P7	77	Male	R	Thalamus	30	0.23 0.35 0.47	589/605	598/302	5°
P8	59	Male	R	Left MCA	32	0.47 0.70 0.93	905/608	600/306	8.5°
P9	52	Male	R	Left MCA	32	0.64 0.96 1.28	903/602	603/302	5°
P10	66	Male	L	Right frontal superior, parietal and posterior area’s	29	0.51 0.76 1.01	908/519	602/299	8.5°
P11	75	Male	R	Left periventricular, temporal and basal ganglia	32	0.63 0.94 1.25	913/497	552/306	5°
P12	49	Male	R	Frontotemporal parietal	33	0.47 0.71 0.95	931/450	303/300	5°

### General paradigm

**Fig. 1:**
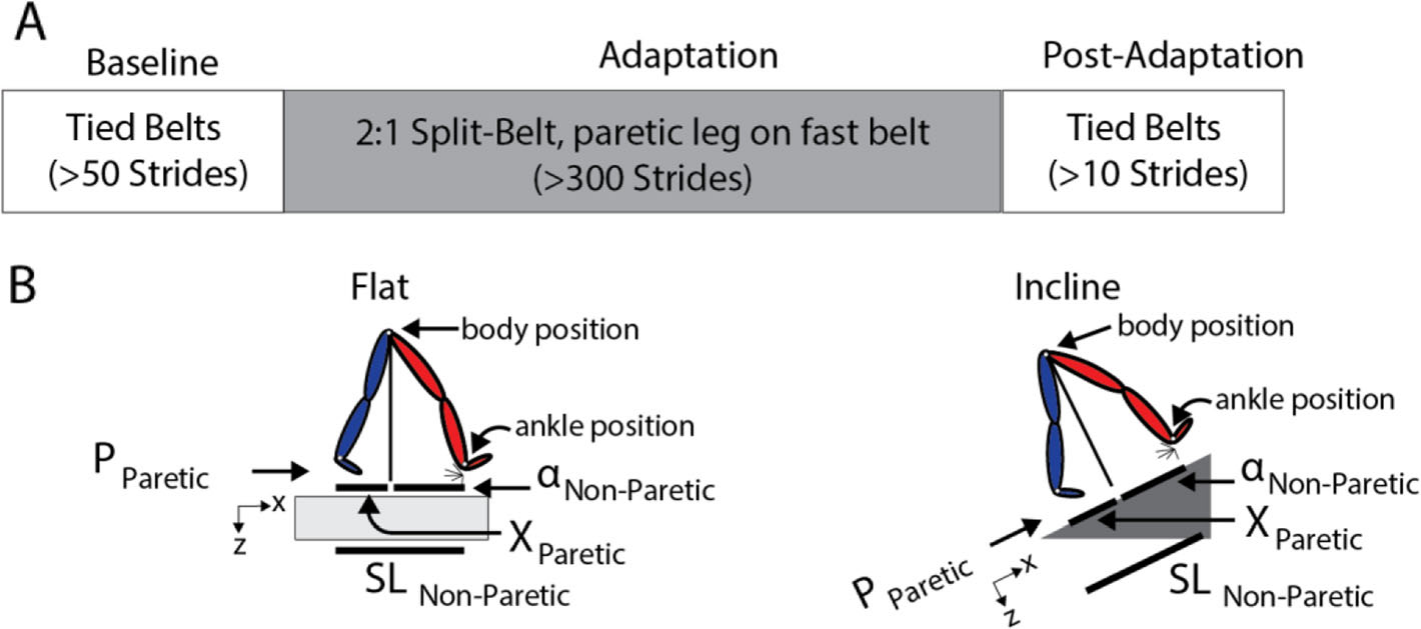
Experimental Paradigm and Kinetic and Kinematic Analysis. **a** Paradigm used for both the flat and incline sessions to assess locomotor adaptation during and after split-belt walking. Subjects walked flat for the entire flat session, and incline (either 5° or 8.5°) for the entire incline session. The walking speeds, duration of epochs, resting breaks and inclination were based on each subject’s ability. **b** The decomposition of step length into leading (α) and trailing (X) leg positions with respect to the body is illustrated for each sloped condition. The body position, which is computed as the average of the greater trochanters, is illustrate with a white dot, and the perpendicular projection of the body onto the surface of the treadmill is illustrated as the black line coming from intersection of the legs. The position of the ankles is illustrated with white dots at the intersection of the foot and shank. This decomposition was done because it is known that inclination affects these aspects of step length differently [15, 16, 46]. Also note that when taking a step, the step length will depend on the position of the leading and trailing leg, which are generating a braking and propulsion force, respectively

All subjects experienced a split-belt protocol while either walking flat or incline throughout two separate experimental sessions (Fig. 1a). The flat session was always performed first. The protocol was tailored (i.e., slope, duration, and speed) to each individual’s ability so that each subject could complete both testing sessions at the same walking speed, given that walking speed directly affects propulsion forces. The subject-specific walking speed on the treadmill (i.e., the mid speed, which is reported in Table 1) was determined by subtracting 0.35 m/s from each subject’s overground walking speed during a Six-Minute Walking Test [64]. We selected this procedure to ensure all individuals completed the entire split-belt walking protocol [29]. The speeds experienced during split-belt walking were selected based on subject’s mid walking speed. The slow speed was defined as 66.6% of the mid speed and the fast speed as 133.3% of the mid speed. These percentages were selected for two reasons: 1) to have a 2:1 split-belt ratio and 2) to have the same averaged speed (i.e., (slow + fast) / 2 = mid speed) throughout all experimental epochs (i.e., baseline, adaptation, and post-adaptation). We selected an inclination of either 5° or 8.5° based on the level of physical fitness of each patient, which was qualitatively assessed with subjects’ self-reported fatigue during the flat session. More specifically, participants reporting fatigue at the end of the flat session were set to walk with a 5° slope during the inclined session, whereas those reporting continued energy at the end of the flat session were set to walk with an 8.5° slope. This was done to ensure that all participants could complete the incline session. Both 5° (e.g., [28, 37, 54, 76]) and 8.5° (e.g., [42, 70]) have been utilized in previous work on the effect of sloped walking on human gait. The flat and incline sessions were separated by 204–360 days without split-belt walking to minimize the effect of multiple exposures to the split-belt environment [45].

Experimental protocols for both sessions consisted of three epochs (i.e., Baseline, Adaptation, and Post-Adaptation). These epochs were used to assess subjects’ baseline walking characteristics and subjects’ ability to adjust and recalibrate their gait for each session-specific slope. Subjects first experienced a baseline epoch, lasting at least 50 strides, to characterize their baseline gait at the specific slope used throughout each session. Subjects walked with both belts moving at the same mid speed (Table 1) in both the flat and incline sessions. A baseline epoch with the belts moving at the slow walking speed (i.e., 66.6% of the mid speed) was also measured during the flat session. However, the slow baseline epoch was removed in the incline session to ensure that all subjects could complete the entire protocol. Next, the Adaptation epoch of at least 300 strides (Table 1) was used to assess subjects’ ability to adjust their gait in response to a split-belt perturbation. During this epoch, the non-paretic leg walked twice as fast as the paretic leg. The paretic leg was defined as contralateral to the lesion site (which was visualized with MRI). The paretic leg always walked on the slow belt in the split-belt condition, even if this experimental design does not always reduce step length asymmetry post-stroke [12, 40, 41, 61, 62]. We chose this experimental design, rather than placing the paretic leg on the fast belt, because inclination augmented the slow leg’s propulsion in young, intact subjects following the split-belt condition [70]. Thus, we focused our study on testing if incline split-belt walking would also augment the paretic propulsion in survivors of a stroke, who have known deficits generating paretic propulsion forces [4, 9]. The duration of the adaptation epoch for each individual is presented in Table 1. We had to tailor the duration of the adaptation epoch based on subject-specific abilities. More specifically, we stopped the adaptation epoch if participants expressed or showed signs of fatigue. That is, we ended the adaptation epoch if participants either had difficulty maintaining their body position in the central region of the treadmill, if their heart rate reached 80% of their maximum heart rate (Max Heart Rate = 220-subject’s age [22, 23]) for 50 consecutive strides, or if participants indicated the desired to stop walking. Despite this variation, all subjects exhibited remarkably similar results. Finally, The Post-Adaptation epoch, lasting at least 10 strides, was used to assess the after-effects when the split-belt condition was removed. Both belts moved at the same mid speed as in the Baseline epoch. We counted the number of strides in real-time to regulate the duration for each epoch, where a stride was defined as the period between two consecutive heel-strikes (i.e., foot landings) of the same leg. All participants took resting breaks as requested, except for the transition from split-to-tied walking where the belts were stopped and restarted as quickly as possible. No steps were taken during the resting breaks; subjects stood or sat still. Also, all subjects wore a safety harness to prevent falls. In addition, there was an instrumented handrail in front of the treadmill for balance support. The handrail data was used to quantify potential differences in handrail holding between the incline and flat sessions. Interestingly, the average force magnitude applied at the handrail was not different between the incline and flat sessions for Baseline (*p* = 0.75), Late Adaptation (*p* = 0.66), or After-Effects (*p* = 0.97). Also, 4 out of 12 individuals that held on to the handrail throughout each experimental session did not qualitatively exhibit smaller changes in adaptation or after-effects across sessions than their counterparts and were, therefore, included in the study.

### Data collection

Kinematic and kinetic data were used to characterize subjects’ ability to adapt their gait during Adaptation, and retain the learned motor pattern during Post-Adaptation. Kinematic data were collected with a passive motion analysis system at 100 Hz (Vicon Motion Systems, Oxford, UK). Subjects’ behavior was characterized with passive reflective markers placed symmetrically on the ankles (i.e., lateral malleolus) and the hips (i.e., greater trochanter) and asymmetrically on the shanks and thighs (to differentiate the legs). The origin of the kinematic data was rotated with the treadmill in the incline conditions such that the z-axis (‘vertical’ in the flat condition) was always orthogonal to the surface of the treadmill (Fig. 1b). Gaps in raw kinematic data were filled with a quintic spline interpolation (Woltring; Vicon Nexus Software, Oxford Uk). Kinetic data were collected with an instrumented split-belt treadmill at 1000 Hz (Bertec, Columbus, OH). Force plates were zeroed prior to each testing session so that each force plate’s weight did not affect the kinetic measurements. In addition, the reference frame was rotated at the session-specific inclination such that the anterior-posterior forces were aligned with the surface on which the subjects walked. A heel-strike was identified in real-time when the raw normal force under each foot reached a threshold of 30 N. This threshold was chosen to ensure accurate counting of strides at all slopes. During data processing we used a threshold of 10 N on median filtered data (with a 5 ms window) to detect the timing of heel strikes more precisely.

### Data analysis

#### Kinematic data analysis

Kinematic behavior was characterized with step length asymmetry, which exhibits robust adaptation in split-belt paradigms (e.g., [60]) and is of clinical interest [31, 32, 59]. It is calculated as the difference in step length between the two legs on consecutive steps (EQ.1). Step length (SL) is defined as the anterior-posterior (i.e., along the x-axis) distance in millimeters between the ankle markers at forward leg heel strike (e.g., paretic step length is defined as the distance between the two ankle markers at the paretic heel strike). Therefore, equal step lengths result in zero step length asymmetry. A positive step length asymmetry indicates that the non-paretic leg’s step length was longer than the paretic leg’s step length. Step length asymmetry was normalized by stride length, which is the sum of two consecutive step lengths, resulting in a unitless parameter that is robust to inter-subject differences in step size. This is particularly relevant when averaging step length asymmetries across subjects since they were walking at different speeds.

1 $$ step\ length\ asymmetry=\frac{SL_{Non- Paretic}-{SL}_{Paretic}}{SL_{Non- Paretic}+{SL}_{Paretic}}=\frac{SL_{Fast}-{SL}_{Slo w}}{SL_{Fast}+{SL}_{Slo\mathrm{w}}} $$

Each step length was also decomposed into anterior and posterior foot distances relative to the hip position (the average of the greater trochanter positions; Fig. 1b) as in previous work [20]. This was done to quantify the leading and trailing legs’ positions relative to the body when taking a step because inclination is known to affect these measures [16, 46]. The leading leg’s position (‘α’) was computed as the anterior-posterior (i.e., along the x-axis) distance in millimeters between the leading leg’s ankle and the hip at heel strike; similarly, the trailing leg’s position (‘X’) was computed as the anterior-posterior (i.e., along the x-axis) distance in millimeters between the trailing leg’s ankle and the hip at heel strike. The hip position, which is a proxy for the body’s position, was estimated as the mean instantaneous position across hip markers. By convention positive α values indicate that the foot landed in front of the hips, whereas negative X values indicate that the trailing leg was behind the hips. Note that the magnitudes of α and X summed to the leading leg’s step length. As indicated in Fig. 1b, α and X were computed aligned to the treadmill’s surface in all sloped conditions.

#### Kinetic data analysis

Kinetic data were used to characterize the adaptation of ground reaction forces (GRF). We focused our analysis on the propulsion component of the anterior-posterior GRF because they are associated with walking speed, hemiparetic severity [9], and step length asymmetry [4]. The anterior-posterior GRF (AP forces) were first low-pass filtered with a cutoff frequency of 20 Hz. Then, forces in Newtons were normalized by each subject’s body weight in kilograms to get a unitless measure that is robust to difference in subjects’ body weight. Similar to previous studies reporting the effect of sloped walking on human gait [28, 42] and previous split-belt studies [53, 57, 70] we computed peak propulsion forces as the maximum AP force (P_Paretic_ and P_Non-Paretic_) excluding the initial positive AP forces following heel strike. Note that we did not remove slope-specific biases due to gravity because we focused on analyzing changes in propulsion forces between epochs of interest. The anterior-posterior kinetic data for one leg for a single subject during the flat testing session was lost due to a hardware malfunction. Thus, analysis of the paretic propulsion forces was performed with 11 subjects rather than 12 subjects.

#### Kinetic and kinematic outcome measures

We computed 5 outcome measures for each kinetic and kinematic parameter: Baseline, Late Adaptation, After-Effects, ΔAdapt, and ΔPost. These 5 outcome measures were computed for the flat session and the incline session. In brief, these outcome measures were used to characterize regular walking (i.e., Baseline) and changes during the Adaptation (i.e., Late Adaptation) and Post-Adaptation epoch (i.e., After-Effects) relative to the Baseline epoch. Finally, we also used these measures to characterize gait changes within either the Adaptation epoch (i.e., ΔAdapt) or the Post-Adaptation epoch (i.e., ΔPost). More specifically, the outcome measure called Baseline was quantified as the average of the last 40 strides of the mid speed Baseline epoch, as in previous studies [27, 29, 70]. This was done to characterize each participants’ gait prior to adaptation in every session. We used these Baseline measures for each session (i.e., the flat and for the incline session) to characterize changes in step length asymmetry and propulsion forces beyond those observed by walking incline (without the split condition). Thus, Late Adaptation was defined as the difference between the average of the last 40 strides of the Adaptation epoch relative to the baseline behavior in each session (i.e., outcome measure called Baseline). The outcome measure called Late Adaptation indicated the steady state behavior reached at the end of the Adaptation epoch and it was characterized with the last 40 strides for consistency with previous work [14, 21, 29, 40, 44, 48, 49, 56, 62, 72]. The outcome measure labelled After-Effects was defined as the difference between the average of the first 5 strides of Post-Adaptation (i.e., Early Post-Adaptation) and the Baseline measure (i.e., early Post-Adaptation - Baseline), as in previous studies [14, 21, 29, 40, 44, 48, 49, 56, 62, 72]. Positive After-Effect values indicated increments in magnitude of a specific parameter during the Post-Adaptation epoch relative to the Baseline epoch, and vice versa for negative values. We also characterized the behavioral changes within Adaptation and Post-Adaptation with ΔAdapt and ΔPost, respectively. ΔAdapt was computed as the difference between Late Adaptation and Early Adaptation (i.e., average of the first 5 strides during the Adaptation epoch, as in previous studies [14, 19, 29, 40, 44, 48, 49, 51, 56, 72]. ΔPost was computed as the difference between Baseline and early Post-Adaptation (e.g., Baseline - Early Post-Adaptation). Baseline was used instead of late Post-Adaptation because the duration of the Post-Adaptation epoch was not sufficiently long in all individuals to extinguish split-belt After-Effects. Thus, Baseline behavior was used a proxy for the late Post-Adaptation behavior. We considered this to be an adequate estimation of the overall changes that occurred in Post-Adaptation, but this is a limitation of our analysis. Therefore, readers should consider the possibility that ΔPost values might be smaller than those reported since people might not return to their baseline behavior after a long period of walking (not recorded). ΔAdapt and ΔPost were calculated such that an increase in the magnitude of a parameter resulted in positive values and vice versa.

### Statistical analysis

A significance level of α = 0.05 was used for all statistical tests, which were performed either with Stata (StataCorp LP, College Station, TX) or with MATLAB (The MathWorks, Inc., Natick, Massachusetts, United States). Normality was assessed with the Lilliefors Test. Except for the step length asymmetries during baseline, all of our step length asymmetry and propulsion outcome measures were normally distributed, thus parametric testing was utilized.

#### Group analyses

We tested the effect of slope on step length asymmetry and propulsion forces. In all statistical analyses we used unbiased values. This was done to identify slope-related differences beyond changes in baseline gait features. We used paired t-tests to compare outcome measures (e.g., ΔAdapt, ΔPost, Late Adaptation, After-Effects) in the flat vs. incline sessions for all gait parameters. We quantified the effect size with a Cohen’s difference, d, which is appropriate for paired data [17]. In addition, we tested if step length values post-adaptation (i.e., ΔPost) were significantly different from zero at each slope with one-sample t-tests, where each *p*-value was corrected for multiple comparisons with Bonferroni corrections.

During baseline walking, it was also of interest to identify differences between the paretic and non-paretic propulsion forces in addition to determining the effect of slope on outcome measures. Therefore, we performed ANOVAs with individual subjects as a random factor to account for the paired nature of the data set and slope and leg as fixed, repeated factors. Effect sizes (*η*^2^) were computed for each factor. These ANOVAs were performed on the peak propulsion values and the trailing leg’s position because our study was focused on the propulsion phase of the gait cycle, which is associated to these two parameters.

The changes of both step lengths during the Adaptation and Post-Adaptation epochs for the flat and incline sessions was also of interest. Therefore, we performed an ANOVA with individual subjects as a random factor to account for the paired nature of the data, and the fixed factors are slope, leg, and epoch. Slope and leg were considered repeated factors in the analysis. Epoch is not repeated and is treated as a between-subject factor given that these epochs are not directly associated [70]. Effect sizes (*η*^2^) were computed for each factor.

#### Regression analyses

We tested the association between leg positions (‘α’ and ‘X’) during speed-specific Baseline and Late Adaptation to determine if Late Adaptation values could be predicted from Baseline values in survivors of a stroke, as observed in young unimpaired subjects [70]. We specifically tested the model |*y|* = a_*_|z|, where *y* is the predicted leg position during Late Adaptation and *z* is the leg position recorded during Baseline. We also tested the ipsilateral association between αs during Late Adaptation and Post-Adaptation and the contralateral association between Xs during these epochs in survivors of a stroke, since these relations were also observed in young, intact individuals [70]. Thus, we tested the model |*y|* = a_*_|z|, where y is each leg’s position during Early Post-Adaptation and z is either the ipsilateral ‘α’ position recorded during Late Adaptation or the contralateral ‘X’ position recorded during Late Adaptation. An absolute value of *z* was utilized so that the data would not bias the results of the regression to be linear by having a cluster of positive (α) and negative (X) data points. Pearson correlation coefficients (r) are presented as an indicator of effect size.

## Results

### Adaptation and recalibration of step length asymmetry are augmented when walking incline

**Fig. 2:**
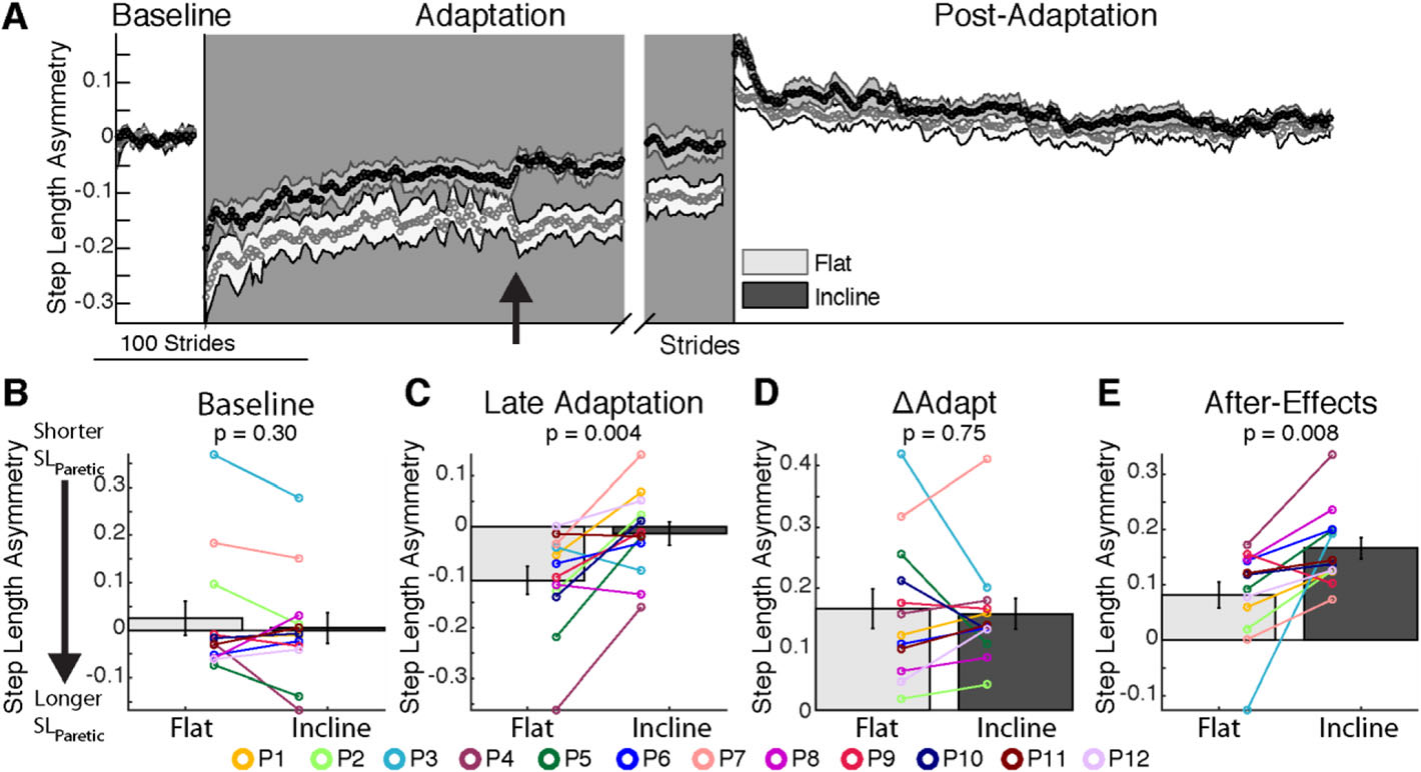
Step Length Asymmetry Adaptation and Recalibration. **a** Stride-by-stride time course of step length asymmetry during Baseline, Adaptation, and Post-Adaptation for each session are shown. Note that each subject’s baseline bias has been removed, resulting in average step length asymmetry values of zero during Baseline. Each data point represents the average of 5 consecutive strides and shaded regions indicate the standard error for each session. For display purposes only, we include in the time courses stride values that were computed with a minimum of 10 subjects and the late adaptation behavior is aligned to the end of each subject’s adaptation epoch. The black arrow indicates a discontinuity in the data caused by many subjects taking a resting break at the same time. **b-e** The height of the bars indicates group average step length asymmetry ± standard errors. Individual subjects are represented with colored dots connected with lines. **b** Baseline: Baseline step length asymmetry is not influenced by slope. **c** Late Adaptation: Note that each session plateaued at different step length asymmetry values during the Adaptation epoch such that subjects reached more symmetric step lengths in the incline session than the flat session (**d**) ΔAdapt: Participants changed their gait by similar amounts during the Adaptation epoch in both sessions. **e** After-effects: Subjects had larger After-Effects during early Post-Adaptation in the incline session than the flat session, which is consistent with the Late Adaptation differences across sessions

Step length asymmetry adaptation and recalibration were augmented by incline walking compared to flat walking. Figure 2a illustrates the evolution of step length asymmetry throughout the flat and incline sessions. Fig. 2b indicates that there was a wide range of individual Baseline step length asymmetries (colored lines, positive values indicate that the non-paretic step length is longer than the paretic step length) and slope did not change the group average biases (*p* = 0.30, t (11) = 1.08, d = 0.15). During Adaptation, participants exhibited similar changes in step length asymmetry from early to late Adaptation (Fig. 2d, *p* = 0.75, t (11) = 0.33, d = 0.08), but they were more symmetric in the incline than the flat session in Late Adaptation (Fig. 2c, *p* = 0.004, t (11) = − 3.68, d = 0.98). Furthermore, the incline session had larger magnitudes of After-Effects during early Post-Adaptation relative to the flat session (Fig. 2e, *p* = 0.008, t (11) = − 3.21, d = 1.08). Thus, incline walking augmented step length symmetry during Late Adaptation and the magnitude of After-Effects.

### Both step lengths contribute to step length asymmetry adaptation and after-effects during incline walking in the asymmetric motor system

**Fig. 3:**
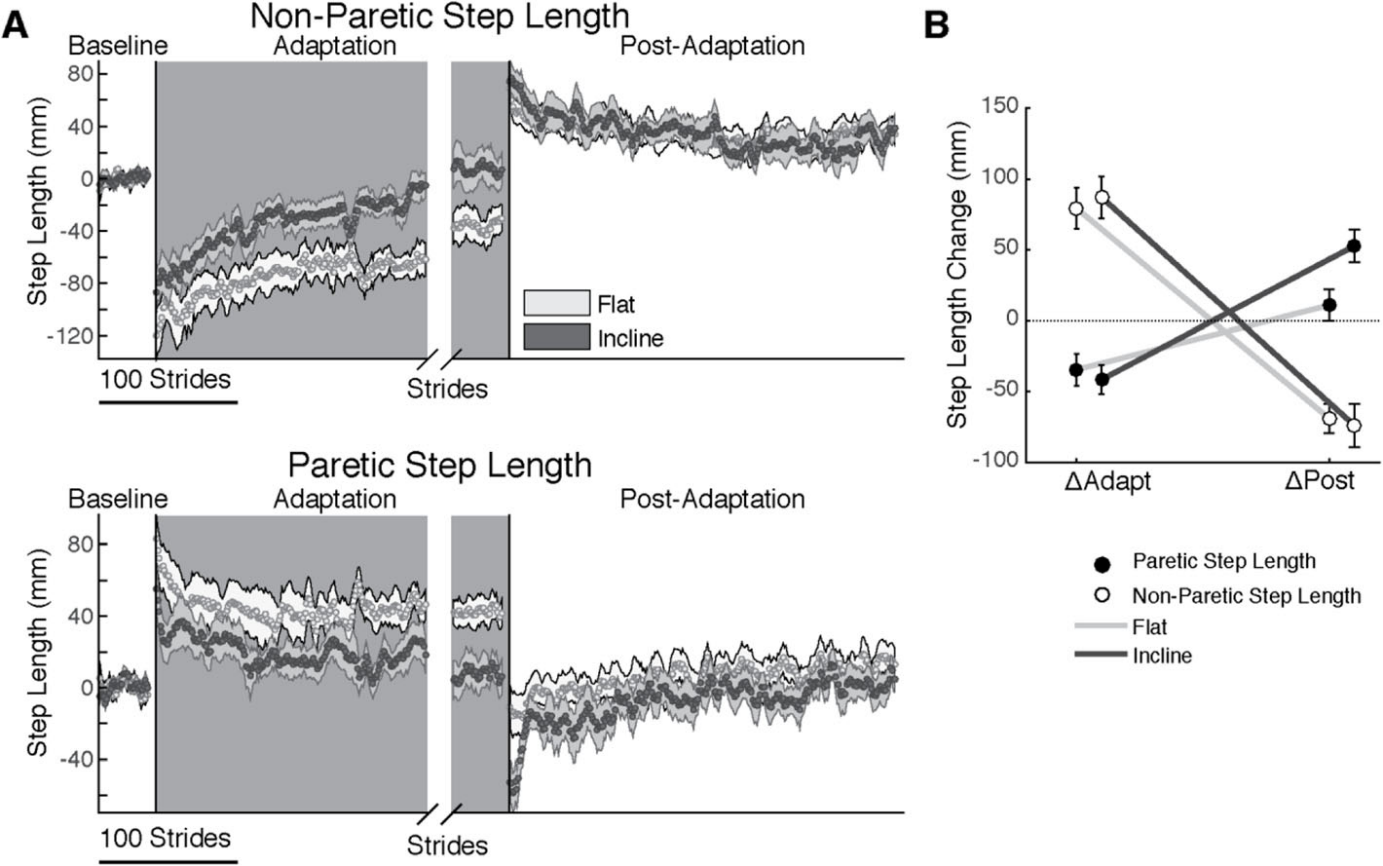
Step length Adaptation and After-Effects. **a** Time courses of step lengths when stepping with either the non-paretic leg (top panel, fast leg during Adaptation) or the paretic leg (bottom panel, slow leg during Adaptation) during three epochs: Baseline, Adaptation, and Post-Adaptation. Note that each subject’s baseline bias has been removed, resulting in average step length values of zero during Baseline. The negative values in the non-paretic step lengths indicate that on average subjects are taking shorter steps with the non-paretic leg relative to baseline walking, whereas the opposite is observed with the paretic one. Each data point represents the average of 5 consecutive steps and shaded regions indicate the standard error for each group. For display purposes only, we include averaged values during Post-Adaptation that were computed with a minimum of 10 subjects and the late adaptation behavior is aligned to the end of each subject’s adaptation epoch. **b** The effect of slope on each leg’s change during Adaptation (ΔAdapt) and Post-Adaptation (ΔPost) is illustrated. Note that both the paretic and non-paretic leg adapted similarly. While the non-paretic leg has recalibrated (ΔPost≠0) following both the flat and incline session, the paretic leg is only recalibrated following incline Adaptation

Survivors of a stroke adjusted both step lengths during split-belt walking. The survivors of a stroke modulate both their slow (paretic) and fast (non-paretic) step lengths during Adaptation and have After-Effects during Post-Adaptation (Fig. 3a). The change of each step length during the Adaptation and Post-Adaptation epochs are quantified in Fig. 3b. There was a significant effect of epoch (p_epoch_ = 0.001, F_epoch_ (1, 22) = 13.54, *η*^2^ =0.38) and interaction between leg and epoch (p_leg#epoch_ < 0.001, F_leg#epoch_ (1, 22) = 94.23, *η*^2^ =0.81) indicating that the step length with the paretic leg is reduced during Adaptation, but increased during Post-Adaptation and vice versa for the non-paretic leg. Overall, slope did not alter step length changes (p_slope_ = 0.16, F_slope_ (1, 22) = 2.14, *η*^2^ =0.09, p_leg_ = 0.44, F_leg_ (1, 22) = 0.61, *η*^2^ =0.03, p_slope#leg_ = 0.18, F_slope#leg_ (1, 22) = 1.88, *η*^2^ =0.08, p_slope#epoch_ = 0.17, F_slope#epoch_ (1, 22) = 1.97, *η*^2^ =0.08), except for the paretic leg’s de-adaptation as quantified by ΔPost (p_leg#epoch#slope_ = 0.016, F_leg#epoch#slope_ (1, 22) = 6.86, *η*^2^ =0.24). More specifically, the paretic step lengths did not exhibit de-adaptation in the flat session (i.e., ΔPost is not different from zero, p_Corrected_ = 1.00, t (11) = 0.92), whereas step lengths for both legs had significant de-adaptation in the incline session (i.e., non-zero ΔPost, p_Corrected_ ≤ 0.005, t (11) ≥ ∣ 4.3∣). Survivors of a stroke use both their paretic and non-paretic leg to counteract the split-belt perturbation and both legs are recalibrated following incline adaptation.

### Slope and speed-specific walking demands determine the distinct step length asymmetries across inclination conditions

**Fig. 4:**
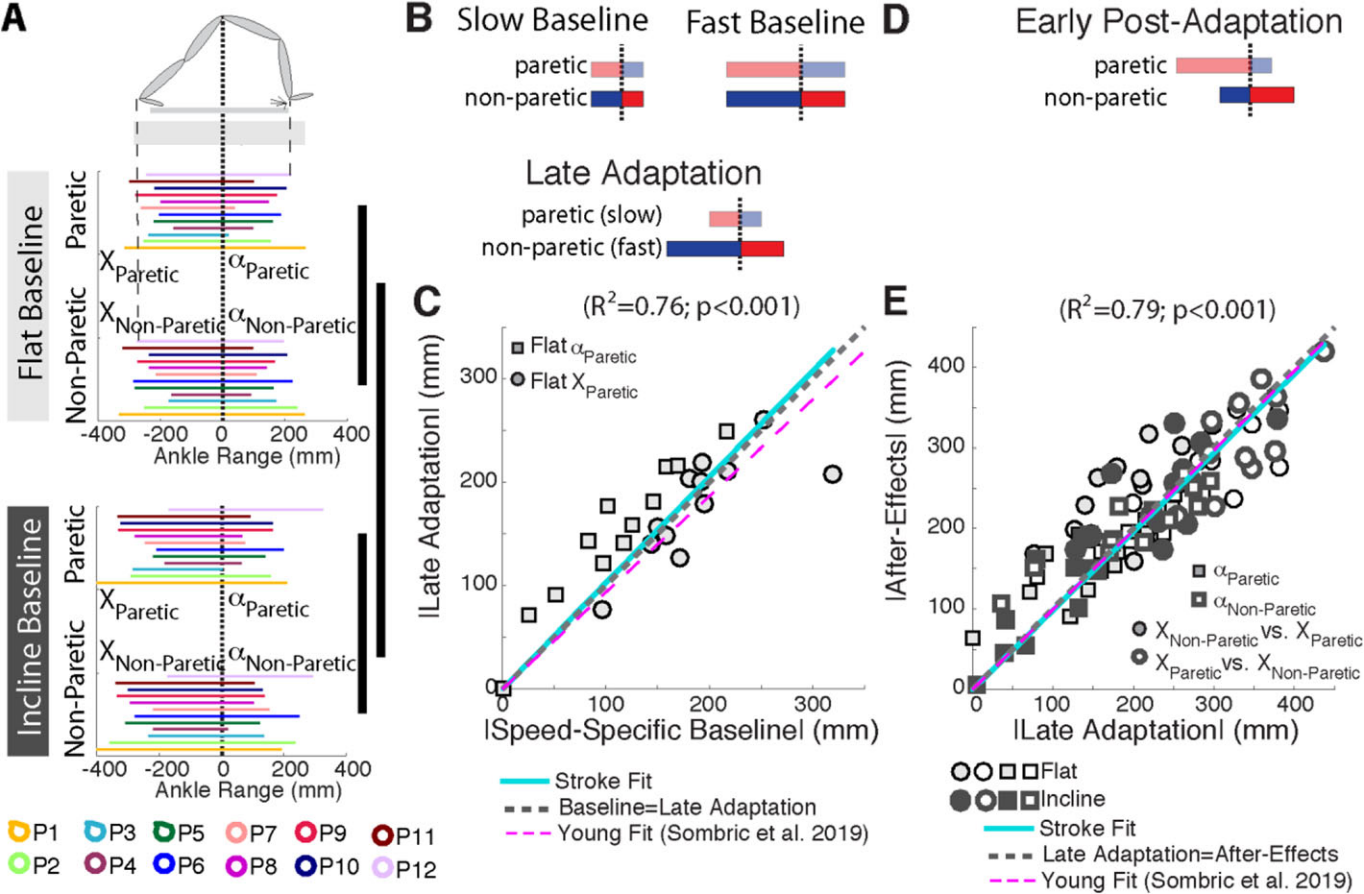
Leg orientation Adaptation and After-Effects. **a** Leg orientations are depicted for individual subjects (as indicated with different colors) in both the flat and incline conditions. Note that subjects orient their legs about their bodies differently and that leg orientations are based on slope. Thick vertical black lines indicated a significant effect of leg (i.e., paretic or non-paretic) and slope (i.e., flat or incline) on trailing leg positions. **b** Schematic of the slow and fast (predicted) baseline behavior for the paretic and non-paretic leg orientations, respectively. The speed-specific leg orientations were regained during Late Adaptation. **c** The similarity between leg orientations across the speed-specific Baseline and Late Adaptation epochs is illustrated by the significant regression (solid cyan line; |*y|* = *a*∗|*x|*, 95% confidence interval for *a* = [0.92, 1.13]). Recall that a slow Baseline was only collected in the flat session, thus only the slow Baseline and Late Adaptation for the paretic leg (which walked slow during Adaptation) are shown. Note that the regression line closely overlaps with the idealized situation in which baseline and late adaptation values are identical (dashed gray line; i.e., *y* = *x*) and the behavior of young, healthy adults ([70], dashed magenta line). **d** Schematic of the leg orientations during early Post-Adaptation. The forward leg positions are ipsilaterally and the trailing leg positions are contralaterally maintained from split-to-tied walking. **e** The ipsilateral and contralateral similarity between α and *X*, respectively, across the Late Adaptation and early Post-Adaptation epochs is quantified with a significant correlation (solid cyan line; |*y|* = *a*∗|*x|*, 95% confidence interval for *a* = [0.94, 1.02]). The idealized situation in which Late Adaptation and early Post-Adaptation values are identical (dashed gray line; i.e., *y* = *x*) and the behavior of young, healthy adults ([70], dashed magenta line) are presented as a reference

Speed and slope-specific leg orientations mediated the distinct step length asymmetries selected during Late Adaptation and early Post-Adaptation. Figure 4a illustrates a top-down view of the baseline leg orientations that contribute to each step length relative to the hips. While we found significantly different leg orientations across individuals (colored lines, p_Individual_ = 0.002, F_Individual_ (11, 11) = 6.99, *η*^2^ =0.87), we observed that the trailing leg position, X, was larger in the incline than flat condition for both legs (p_Slope_ = 0.042, F_Slope_ (1, 11) = 5.32, *η*^2^ =0.33, p_Leg_ = 0.22, F_Leg_ (11, 11) = 1.72, *η*^2^ =0.14, p_Slope#Leg_ = 0.76, F_Slope#Leg_ (1, 11) = 0.10, *η*^2^ =0.01). The schematic in Fig. 4b illustrates the relation between baseline speed-specific leg orientations and late Adaptation [70]. Figure 4c indicates that the participants’ leg orientations during slow Baseline walking predict well those achieved during late Adaptation (solid cyan line; |y| = a*|x|; 95% confidence interval of a = [0.92, 1.13], t_slope_ (95) = 20.0, R^2^ = 0.76, *p* < 0.001, r = 0.84). We also show as a reference, the relation between (recorded) Baseline and (predicted) Late Adaptation leg orientation values for both legs and both inclinations in young unimpaired individuals [70] (magenta dashed line; (|y| = a*|x|; 95% confidence interval of a = [0.91, 0.96], t_slope_ (95) = 73.04, R^2^ = 0.89, *p* < 0.001, r = 0.94). Note the similarity between the intact and lesioned behavior (cyan vs. magenta lines).

Moreover, we found that the leg orientations achieved during Late Adaptation were predictive of subjects’ Post-Adaptation behavior (Fig. 4d). Specifically, the leading leg’s orientations were similar before and after removal of the split-belt perturbation (i.e., Late Adaptation α_Paretic_ = Post-Adaptation α_Paretic_ and vice versa) whereas the trailing legs’ orientations were swapped between the legs (i.e. Late Adaptation X_Paretic_ = Post-Adaptation X_Non-Paretic_ and vice versa). This is supported by the significant relationship between Late Adaptation and Post-Adaptation leg orientations observed when individual subjects’ values for each leg and both sloped sessions are regressed (Fig. 4e; solid cyan line; |y| = a*|x|; 95% confidence interval of a = [0.94, 1.02], t_slope_ (95) = 47.5, R^2^ = 0.79, *p* < 0.001, r = 0.86). We also show as a reference, the relation between (recorded) Late Adaptation and (predicted) Post-Adaptation leg orientation values for both legs and both sloped conditions in young, intact individuals (magenta dashed line; (|y| = a*|x|; 95% confidence interval of a = [0.95, 1.03], t (95) = 47.54, R^2^ = 0.78, *p* < 0.001, r = 0.87). Note the similarity between the intact and lesioned behavior (cyan vs. magenta lines). Similar to the intact motor system, the lesioned motor system is able to recover speed and slope-specific leg orientations during Late Adaptation, which predict after-effects during Post-Adaptation.

### Larger after-effects of propulsion forces following split-belt incline walking

**Fig. 5:**
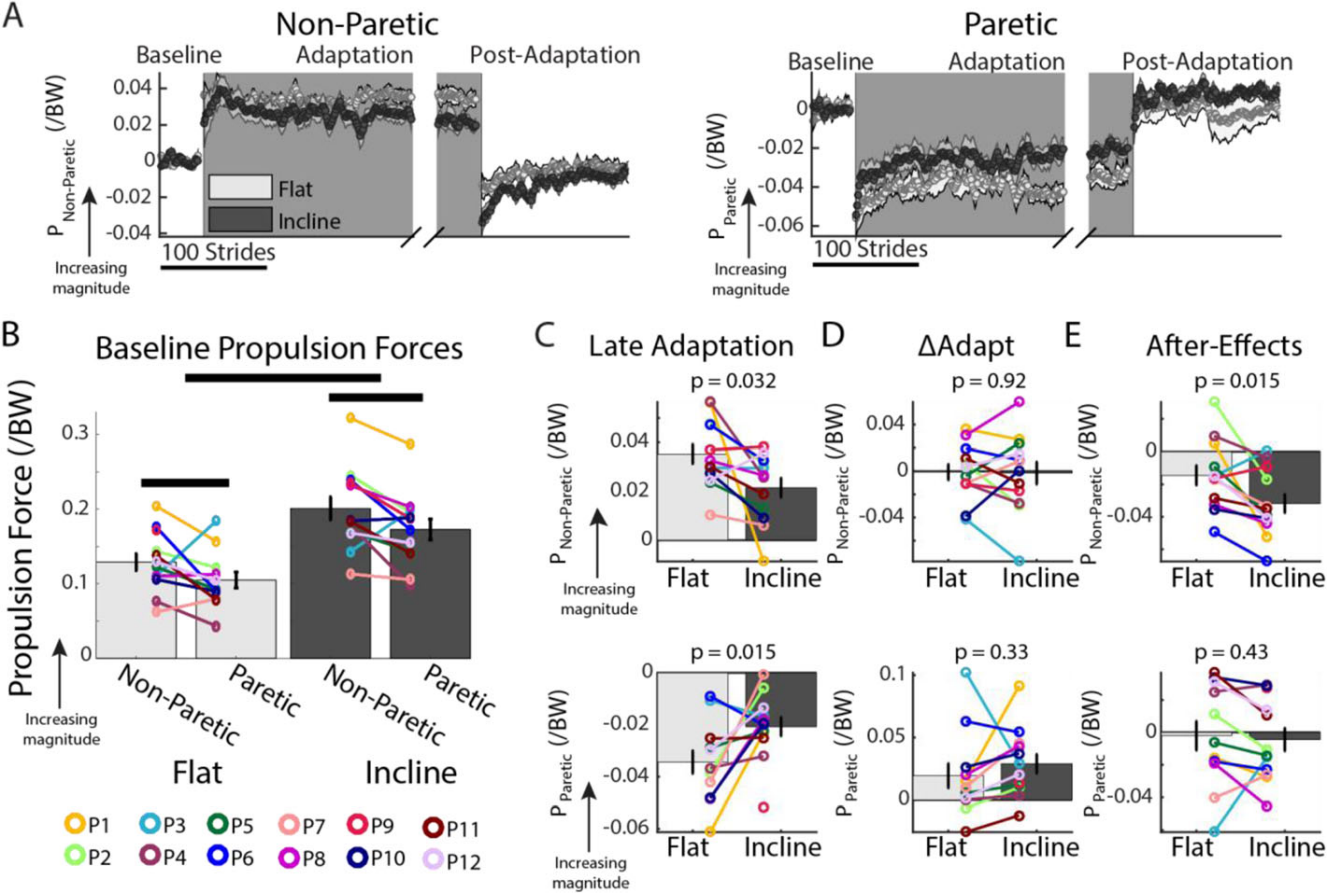
Propulsion force Adaptation and After-Effects. **a** Stride-by-stride time courses of propulsion forces of the non-paretic (top panel) and paretic leg (bottom panel) are shown during self-selected Baseline, Adaptation, and Post-Adaptation. Note that each subject’s baseline bias has been removed, resulting in average propulsion values of zero during Baseline. Each data point represents the average of 5 consecutive strides and shaded regions indicate the standard error for each group. For display purposes only, we include stride values during Post-Adaptation that were computed with a minimum of 10 subjects and the late adaptation behavior is aligned to the end of each subject’s adaptation epoch. **b-e** We display group average values for propulsion force outcome measures ± standard errors. Individual subjects are represented with colored dots connected with lines. **b** Baseline: Thick horizontal black lines indicated that there is a significant effect of leg (i.e., paretic or non-paretic) and slope (i.e., flat or incline) on propulsion forces. On average, stroke subjects generate larger propulsion forces with their non-paretic leg, and they generate larger propulsion forces with both legs when walking incline. However, some individual stroke subjects generate larger propulsion forces with their paretic than their non-paretic leg. **c** Late Adaptation: Stroke subjects were closer to their baseline propulsion forces in the incline than the flat sessions. Moreover, baseline propulsion forces in the incline session were larger than the flat session (Fig. 2c). Taken together, these results suggest that stroke subjects are forced to propel more during incline split-belt walking with both legs compared to flat split-belt walking. **d** ΔAdapt: Propulsion forces were similarly modulated during the Adaptation epoch for both sloped conditions. **e** After-Effects: Even though both sloped sessions did not change the extent of propulsion force adaptation (ΔAdapt), slope influenced the After-Effects for the non-paretic leg, but not the paretic leg

Sloped walking influenced the extent of recalibration of the non-paretic propulsion forces. Figure 5a shows that propulsion forces were altered during the Adaptation epochs. These data are plotted relative to Baseline propulsion forces (i.e., mid speed), which were larger in the incline condition and the non-paretic leg for both sloped conditions (Fig. 5b: p_Individual_ = 0.007, F_Individual_ (11, 10) = 4.84, *η*^2^ =0.83, p_Slope_ < 0.0001, F_Slope_ (1, 10) = 4.84, *η*^2^ =0.85, p_Leg_ = 0.040, F_Leg_ (1, 10) = 5.42, *η*^2^ =0.33, p_Slope#Leg_ = 0.43, F_Slope#Leg_ (1, 10) = 0.69, *η*^2^ =0.06). Note that subjects were closer to generating Baseline-like propulsion forces during Late Adaptation in the incline session compared to the flat session for both legs, resulting in larger Late Adaptation paretic propulsion forces in the incline session (Fig. 5c). Even though the Late Adaptation behavior was different across sessions (Fig. 5c; non-paretic propulsion: *p* = 0.032, t (11) = 2.46, d = 0.98, paretic propulsion: *p* = 0.015, t (10) = − 2.94, d = 1.23), the changes in propulsion forces from early to late Adaptation were similar across sloped conditions (Fig. 5d; non-paretic propulsion: *p* = 0.92, t (11) = 0.10, d = 0.02, paretic propulsion: *p* = 0.33, t (10) = − 1.04, d = 0.33). While paretic propulsion After-Effects are similar in either sloped conditions (Fig. 5e, *p* = 0.43, t (10) = .82, d = 0.17), the non-paretic After-Effects are larger in magnitude following incline adaptation (*p* = 0.015, t (11) = 2.90, d = 0.82). Note that the paretic propulsion forces change the most during Adaptation (Fig. 5d), whereas the non-paretic propulsion forces are the ones exhibiting after-effects during Post-Adaptation (Fig. 5e). In summary, incline walking results in augmented paretic propulsion forces during Adaptation and reduced non-paretic propulsion force After-Effects.

## Discussion

### Summary

We investigated the effect of locomotor propulsion demands on motor adaptation and recalibration of gait in the asymmetric motor system by altering the slope of the split-belt walking surface (i.e., flat vs. incline conditions). Survivors of a stroke adapted their step length asymmetry more in the incline than the flat condition resulting in Late Adaptation step length asymmetries that were smaller in magnitude (i.e., more successfully recovered baseline step length asymmetry). We also found that the speed-specific leg orientations (i.e., α and *X*) for both legs during Adaptation were predictive of those Post-Adaptation, leading to greater step length asymmetry after-effects in the incline than flat sessions. Lastly, larger step length asymmetry after-effects resulted from shorter paretic step lengths and lower non-paretic propulsion forces during Post-Adaptation in the incline session. In summary, the ability to control leg orientation to meet speed and force demands during split-belt walking is maintained post-stroke, which can be exploited for designing effective gait rehabilitation interventions.

### Post-stroke gait adapts more in response to larger propulsion demands

We found that survivors of a stroke behaved similarly to young, intact adults in their response to sloped split-belt walking [70]. Specifically, survivors of a stroke were able to augment their propulsion forces in response to incline split-belt walking as observed in young, healthy adults [70]. It should be noted that all our analysis was done with peak forces, but we found similar effects with other metrics to quantify propulsion, such as impulse or mean force (data not shown). Our observation is consistent with previous literature indicating that patients in the chronic phase post-stroke can modulate paretic propulsion forces in response to task demands [3, 24–26, 34]. In particular, we observed during split-belt walking (i.e., adaptation period) that individuals exhibited larger non-paretic and paretic propulsion forces relative to baseline and early adaptation, respectively. These increments are possibly enabled by augmenting latent central drive to plantarflexors [2]. Interestingly, we find significant reduction in the fast (i.e. non-paretic) leg’s propulsion forces post-adaptation (i.e., after-effects) relative to baseline walking. This finding is consistent with our previous study in young, unimpaired adults [70], but not with other studies reporting no significant changes in propulsion forces following split-belt walking in the flat condition [57, 66]. We speculate that this might be because we use more naturalistic walking speeds than in previous experimental designs [57, 66].

Our results provide further evidence that the adaptation of step length asymmetry can be predicted from Baseline walking. Notably, it has been previously suggested that patients’ gait asymmetries during baseline walking determine the extent to which they can adapt their movements in the unusual split condition [50]. We observed that survivors of a stroke reach distinct asymmetry levels across the incline and flat conditions. Thus, our results support previous findings [40, 41] indicating that it is not baseline gait asymmetry, but kinetic demands that govern the degree to which patients adapt their motor patterns in the split-belt task. More specifically, survivors of a stroke adjusted their leg orientations to augment the propulsion forces required for walking in the incline split condition as observed in young, healthy adults [70]. In other words, the forces generated to propel one’s body forward constitute an important control variable regulating the adaptation of movements in the intact and asymmetric motor systems. Further, a recent study suggests that individuals adjust leg orientations to harness energy from the treadmill in the split condition [67]. While this theory explains well the orientation of the leading leg, it does not match well the observed orientation of the trailing leg (Additional file). Perhaps, other factors, such as stability [10], also contribute to the control of leg orientation in walking. It should be pointed out that we only tested the relation between leg orientations during baseline and adaptation in the paretic leg. We speculate that the same would have been observed in both legs and sloped conditions like young adults [70]. This is a reasonable expectation given that survivors of a stroke exhibited similar control of leg orientations to young adults during Late Adaptation and early Post-Adaptation for both legs and sloped conditions. Nonetheless, future work is needed to verify that the relation between baseline and adaptation is observed for distinct inclination conditions, or if the legs were walking at different belt-speed ratios than the ones we used.

### Bilateral adaptation in survivors of a stroke contrasts unilateral adaptation in young adults

Survivors of a stroke recruited both legs in order to adapt their gait, whereas young adults primarily adapted one leg. Notably, we observed that survivors of a stroke adapted both the paretic (slow belt) and non-paretic (fast belt) step lengths, whereas we previously found that young individuals predominantly adjusted the fast belt step length [60, 70]. This could be because survivors of a stroke may require more repetitions in the altered environment [75] to recover their baseline leg orientation with their paretic leg, whereas intact subjects can do so immediately after the split condition is introduced. Alternatively, it could be that the larger neural coupling post-stroke [35] enhances bilateral adaptation. In other words, it might not be possible for individuals who had experienced a stroke to adapt one leg in isolation due to neural drive sent to both limbs. Regarding post-adaptation, paretic after-effects were only observed in the incline condition. More specifically, paretic step lengths become longer than in baseline walking, which may be beneficial for survivors of a stroke who take short paretic step lengths [4]. On the other hand, non-paretic after-effects were observed regardless of the sloped condition. This was atypical since the non-paretic leg walked fast in the split condition and young adults only exhibit after-effects in the leg that walked slow [70]. Thus, it was unexpected to observe non-paretic step lengths shorter than those taken during baseline. This shortening of non-paretic step lengths might be a strategy to recover balance (e.g., [18]), which is challenged upon removal of the split condition [10, 29]. In summary, survivors of a stroke adapt both legs during split-belt walking, but paretic step length after-effects are only observed following incline split-belt walking.

### Neurorehabilitation through reinforcement of a corrective pattern during adaptation, rather than short-lived after-effects post-adaptation

The long-term therapeutic effect of locomotor adaptation with split-belt treadmills may be due to walking with the motor demands of the split-belt task, rather than the adaptation effects observed post-adaptation. Split-belt walking has been shown to reduce long term gait asymmetry [7, 47, 61]. Little is known about the aspects of split-belt walking that underlie these long-term changes: the cumulative effect of brief after-effects post-adaptation or the repeated exposure to motor demands specific to the split condition during the adaptation period. After-Effects could lead to motor improvements [5] such as temporarily reduced gait asymmetry [12, 62]. However, these after-effects are short lived and decrease as individuals experience multiple days of practicing the split-belt condition [38, 45, 71]. It is known that regular treadmill walking cannot modify gait asymmetries post-stroke [33, 58, 69], suggesting that the specific motor demands of the split-belt task might be important for neurorehabilitation. For example, we observe that the split condition (during the adaptation period) forces patients to take longer paretic step lengths and generate greater paretic propulsion forces. Perhaps practice of these gait features through multiple exposures to the split situation reinforces those patterns and ultimately leads to long-term reductions of gait symmetry overground. It is also possible that the strenuous nature of split-belt walking increases neural plasticity, as shown with other high-intensity exercises [1]. Thus, incline split-belt walking may be beneficial not only for inducing greater paretic propulsion, but also because it is more demanding than level walking [30]. Lastly, the initial disruption of step length asymmetry might trigger exploration of new locomotor patterns [52] that could converge to more metabolically efficient gait than their baseline walking pattern [67, 68]. In summary, the long-term benefit of split-belt walking may originate from practicing and reinforcing motor patterns imposed by the split condition, rather than repeating the briefly lived after-effects.

### Clinical implications

Split-belt walking has been shown to induce long term changes that could improve the mobility of those who have had a stroke [7, 47, 61]. Previous studies have investigated the impact of training design (e.g., paretic leg on the slow belt vs. fast belt) on the therapeutic effect of split-belt walking [40, 61]. Our work contributes to this literature by indicating that this choice depends on the rehabilitation outcome of interest. For instance, our results suggest that placing the paretic leg on the fast belt would force subjects to augment their paretic propulsion forces and lengthen their paretic steps during split-belt walking, which could be advantageous to some patients. This idea is consistent with a pilot study showing that platarflexor moments trained on the fast side increase following multiple split-belt walking sessions [8]. This therapeutic effect is beneficial to individuals with baseline asymmetries due to shorter paretic step lengths coupled with plantar flexor weakness [6]. It remains, however, an open question the extent to which incline split-belt walking could augment paretic propulsion when the paretic leg is placed on the fast belt. Future studies are needed to test this given the limited changes in paretic propulsion that we observed post-adaptation compared to controls [70]. In sum, our study provides greater understanding of the motor demands associated to the split-belt task, which could be harnessed for gait neurorehabilitation.

Incline split-belt training may be a promising way to augment locomotor and adaptation and recalibration in the lesioned motor system. Not everyone who has had a stroke re-learns to walk symmetrically following several weeks of flat split-belt training [7, 47, 61]. Thus, it is clinically relevant to explore alternative strategies to augment adaptation in survivors of stroke other than increasing the speed difference [7, 77] since not all patients can walk with large speed differences. While this work indicates that adaptation can be augmented in patients, previous work indicates that overground walking post-stroke is most improved following decline, rather than incline, interventions [11]. Moreover, it has been previously observed that motor patterns observed on the treadmill do not fully transfer to overground walking [63, 71, 73, 74]. Thus, future studies are needed to determine if the augmented adaptation in the incline environment transfers to flat overground walking. Furthermore, we designed our study to evaluate changes in step length asymmetry relative to baseline walking. It is, however, clinically relevant to determine if the augmented adaptation and after-effects lead to reduced step length asymmetry in absolute terms. Finally, it must be noted that participants were moderately to mildly impaired (i.,e, 21 < Fugl-Meyer Assessment leg motor score ≤ 34). Therefore, it remains an open question if similar results could be observed in more severely affected individuals or with different demographics than our cohort of participants.

## Conclusion

We investigated the influence of augmenting propulsion demands during walking on the plasticity of locomotion post-stroke. We found that individuals who have suffered a stroke adapt their gait more during split-belt walking and have greater after-effects post-adaptation when propulsion demands are increased by inclining the treadmill. Like intact subjects, after-effects are predicted by each participant’s leg orientations achieved during split-belt walking, which in turn is predicted by subject-specific leg orientations during baseline walking. These results have two implications. First, these findings indicate that survivors of a stroke can adjust their movements to meet kinetic demands imposed by the walking condition. Second, subject-specific baseline behavior can predict the extent to which people will adjust their movements during and after the split condition. Taken together, our findings contribute to existing literature investigating mechanisms underlying locomotor adaptation induced by split-belt walking, which could be exploited for designing effective gait rehabilitation interventions post-stroke.

## Supplementary information

**Additional file 1:Figure S1.** Adaptation and After-Effects of the leading leg and training leg positions. (A) Stride-by-stride time courses of leg positions (α and X) for the non-paretic and paretic leg are shown during self-selected Baseline, Adaptation, and Post-Adaptation. Each data point represents the average of 5 consecutive strides and shaded regions indicate the standard error for each group. The beginning and Late Adaptation group average behavior are shown for the Adaptation epoch. For display purposes only, we include stride values during Post-Adaptation that were computed with a minimum of 10 subjects. (B) Schematic of the self-selected Baseline, early Adaptation, and late Adaptation behavior for the paretic and non-paretic leg orientations, respectively. Note that during the adaptation period, the leading positions (α) increase for both legs, whereas the training position (X) increases for the paretic leg (slow leg) and drecreases for the non-paretic leg (fast leg).

## Data Availability

The datasets used and/or analyzed during the current study are available at https://osf.io/vm597/.

## Abbreviations

SL: step length
α: leading leg position relative to the hip at leading leg heel strike
X: trailing leg position relative to the hip at leading leg heel strike
GRF: ground reaction forces
AP: anterior-posterior
P_Pareitc_: paretic peak propulsion forces
P_Non-Pareitc_: non-paretic peak propulsion forces
MRI: magnetic resonance imaging
