# Supplementary material for: Augmenting propulsion demands during split-belt walking increases locomotor adaptation of asymmetric step lengths
Source: J Neuroeng Rehabil. 2020 Jun 3;17:69. doi: 10.1186/s12984-020-00698-y (PMC7268294; doi:10.1186/s12984-020-00698-y)
Supplement: Supplementary file 1 — Additional file 1:Figure S1. Adaptation and After-Effects of the leading leg and training leg positions. (A) Stride-by-stride time courses of leg positions (α and X) for the non-paretic and paretic leg are shown during self-selected Baseline, Adaptation, and Post-Adaptation. Each data point represents the average of 5 consecutive strides and shaded regions indicate the standard error for each group. The beginning and Late Adaptation group average behavior are shown for the Adaptation epoch. For display purposes only, we include stride values during Post-Adaptation that were computed with a minimum of 10 subjects. (B) Schematic of the self-selected Baseline, early Adaptation, and late Adaptation behavior for the paretic and non-paretic leg orientations, respectively. Note that during the adaptation period, the leading positions (α) increase for both legs, whereas the training position (X) increases for the paretic leg (slow leg) and drecreases for the non-paretic leg (fast leg). [file 12984_2020_698_MOESM1_ESM.docx]

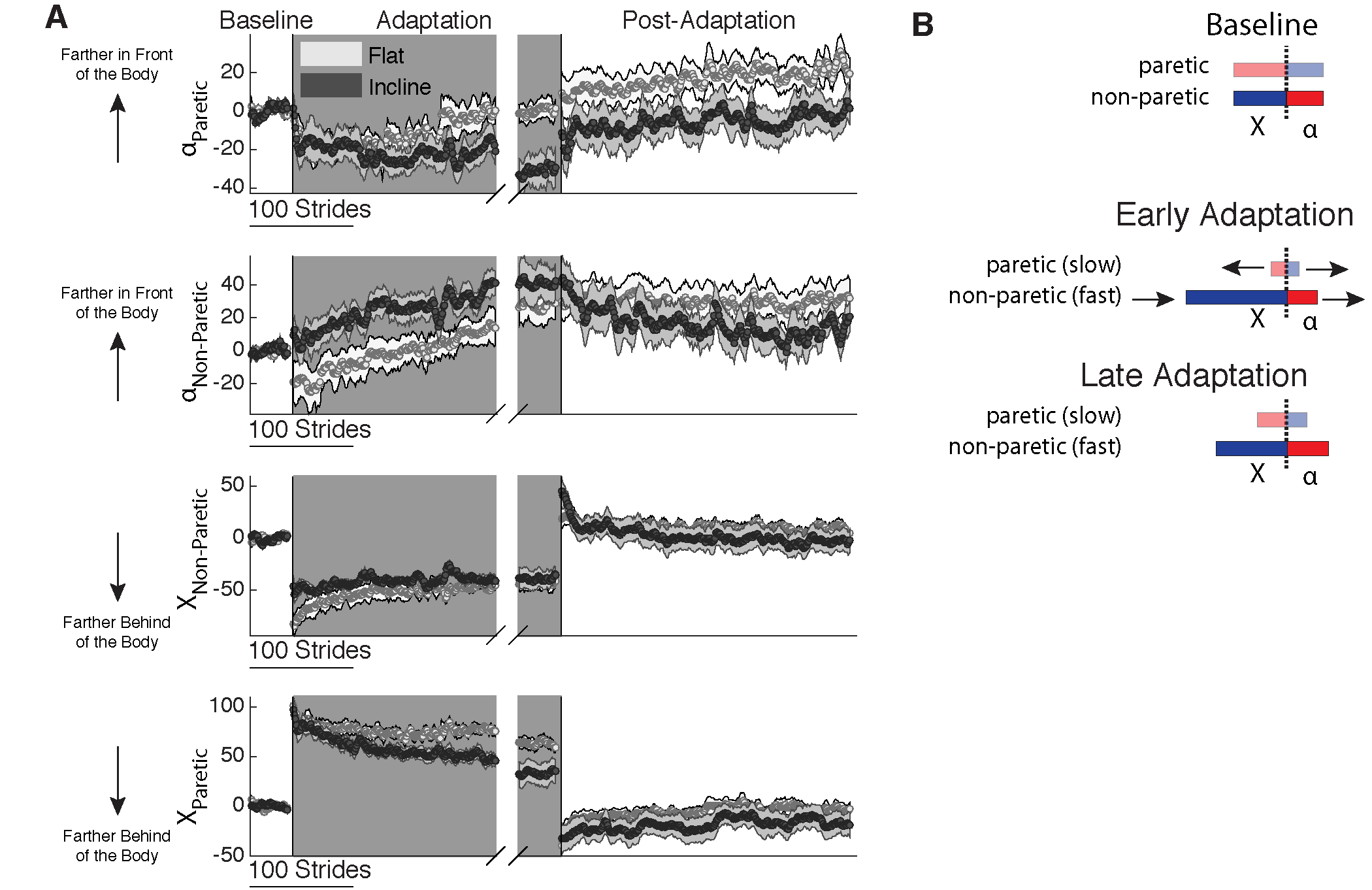


**Supplementary Figure 1: Leg Position Adaptation and After-Effects| (A)** Stride-by-stride time courses of leg positions (α and X) for the non-paretic and paretic leg are shown during self-selected Baseline, Adaptation, and Post-Adaptation. Each data point represents the average of 5 consecutive strides and shaded regions indicate the standard error for each group. The beginning and Late Adaptation group average behavior are shown for the Adaptation epoch. For display purposes only, we include stride values during Post-Adaptation that were computed with a minimum of 10 subjects. **(B)** Schematic of the self-selected Baseline, early Adaptation, and late Adaptation behavior for the paretic and non-paretic leg orientations, respectively. Note that there is a general forward movement of the leg position of the non-paretic leg, but the paretic leg increases both the leading and trailing positions.
